# The rapid improvement in visual field defect observed with weekly perimetry during intensity-modulated radiotherapy for optic nerve sheath meningioma

**DOI:** 10.1007/s13691-019-00371-9

**Published:** 2019-04-06

**Authors:** Toshihiko Inoue, Osamu Mimura, Koji Ikenaga, Yoshishige Okuno, Iku Nishiguchi

**Affiliations:** 1 Ashiya Radiotherapy Clinic Nozomi, 3-84 Yoko-cho, Ashiya, Hyogo 659-0034 Japan; 2 Department of Neuro-ophthalmological Therapeutics, Hyogo College of Medicine, 1-1 Mukogawa-cho, Nishinomiya, Hyogo 663-8501 Japan

**Keywords:** Optic nerve sheath meningioma (ONSM), Intensity-modulated radiotherapy (IMRT), Humphrey automated perimetry, Rapid improvement of visual field defect

## Abstract

During precision radiotherapy to treat optic nerve sheath meningioma, early improvement in visual function has been seen. This has been difficult to explain biologically. In the present study, we aimed to investigate this rapid improvement in visual function. To this end, we prospectively tested a single patient’s visual field (VF) using Humphrey automated perimetry at weekly intervals. The patient exhibited significant stepwise improvement in VF during an intensity-modulated radiotherapy course.

## Introduction

Optic nerve sheath meningioma (ONSM) is a rare benign tumor that compromises optic nerve function by compressing the pial vascular supply. We previously reported that early intervention with precision radiotherapy to treat ONSM resulted in a rapid and complete reversal of visual impairment [1]. However, this phenomenon is difficult to explain biologically [2]. In the present study, we aimed to investigate this rapid improvement in visual field (VF) defect by prospectively repeating Humphrey automated perimetry (HAP) testing at weekly intervals.

## Case report

**Fig. 1:**
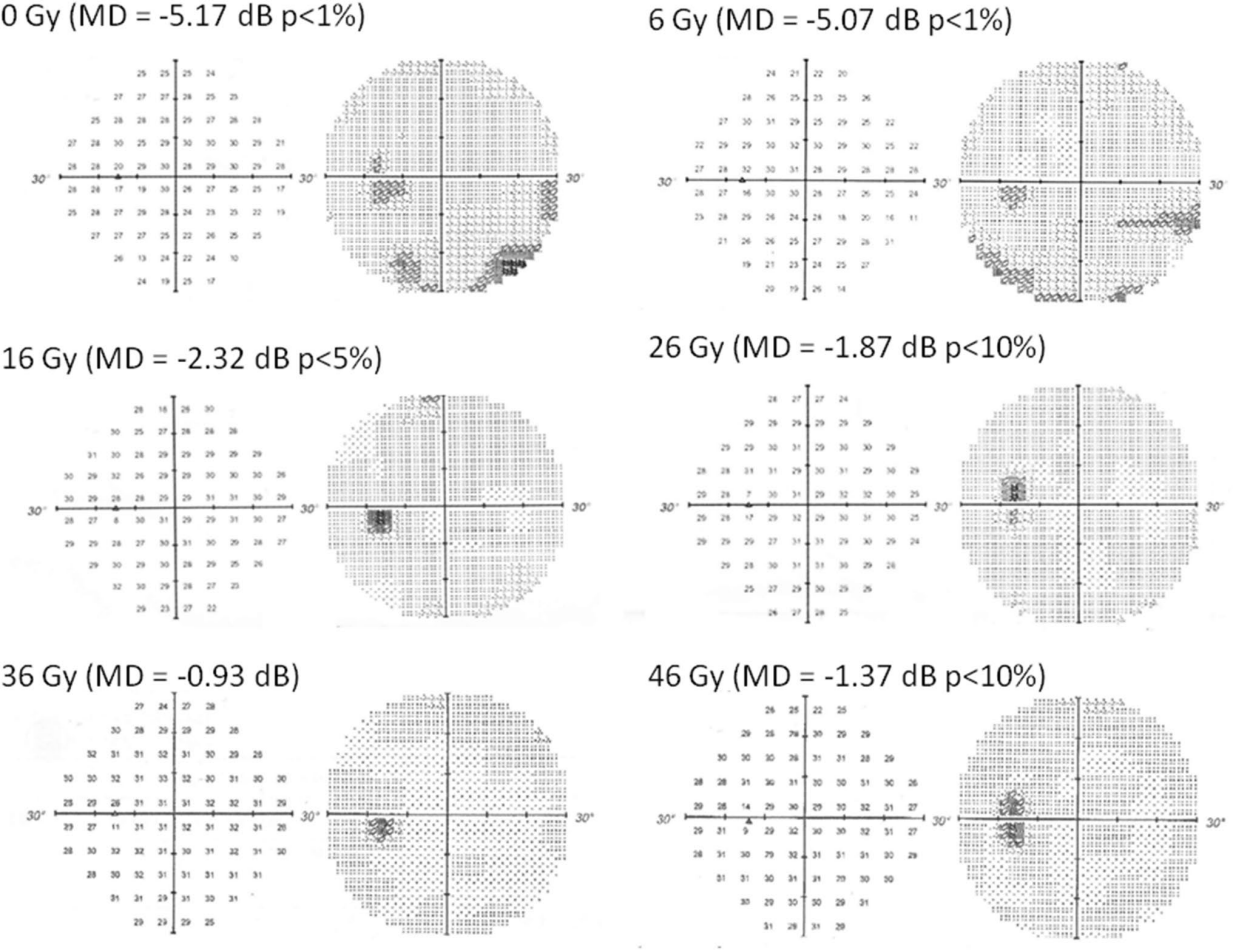
A 40-year-old male patient had a low-grade inferior altitudinal defect. Repeated weekly Humphrey automated perimetry showed that the apparent inferior altitudinal visual field defect disappeared rapidly after irradiation of 16 Gy, and the stepwise improvement of mean deviation occurred in visual field during intensity-modulated radiotherapy

**Fig. 2:**
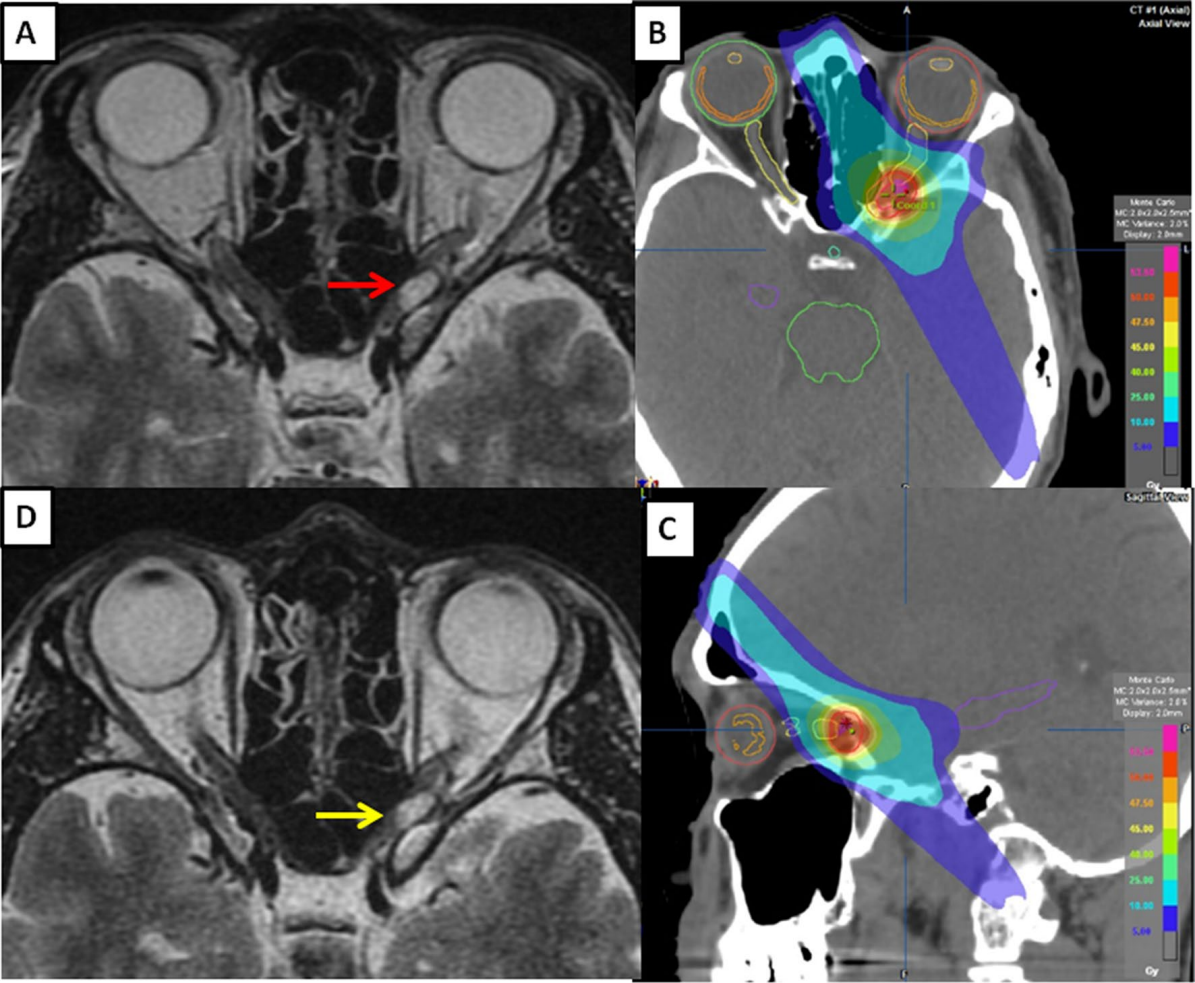
Pretreatment magnetic resonance imaging (MRI) showed a fusiform type of intraorbital tumor (red arrow) at the pre-canalicular portion of the left optic nerve (**a**). The patient underwent intensity-modulated radiotherapy (IMRT) at a dose of 50 Gy delivered in 25 fractions over 35 days in May 2018 (**b, c**). Despite a rapid improvement in visual field, detected with Humphrey automated perimetry, MRI did not show any significant change in tumor size (yellow arrow) during the early stages of IMRT (**d**)

A 40-year-old man with a 3-month history of left VF impairment was referred to the Department of Ophthalmology at Hyogo College of Medicine in April 2018. His logarithm of the minimum angle of resolution (logMAR) corrected visual acuity (VA) was − 0.18, which converts to 1.5 in decimal notation. He showed a slightly decreased light reflex and a relative afferent pupillary defect. Optical coherence tomography revealed that his left circumpapillary retinal nerve fiber layer was slightly thinner than normal, while HAP showed a low-grade inferior altitudinal defect with a mean deviation (MD) of − 5.24 dB (*p* < 1%: Fig. 1). Magnetic resonance imaging (MRI) showed widening and winding of the optic nerve (ON) with tram-track sign and a fusiform tumor measuring 10 × 6 × 8 mm (volume 0.35 cm^3^). The tumor was located on the left distal ON at the precanalicular portion (Fig. 2). The patient was, therefore, diagnosed with ONSM.

He underwent 7-beam IMRT in May 2018, with a prescribed total dose of 50 Gy delivered in 25 fractions over 35 days without steroid medication before and during the precision radiotherapy. He was treated using a 6-MV X-ray Novalis unit™ (BrainLAB AG, Munich, Germany). With regard to treatment planning, we aimed to make the planning target volume as small as possible. The gross tumor volume (GTV) was determined using computed tomography and MRI fusion imaging. This ensured accuracy in GTV delineation (Fig. 2b, c). The tumor volume was calculated from contrast-enhanced 3-dimensional MRI with treatment planning carried out using i-PLAN Dose ver. 4.1.2™ (BrainLAB AG). We defined the GTV as the clinical target volume (CTV), and the CTV as the internal target volume (ITV). The planning target volume (PTV) was defined as the ITV plus the setup margin (SM) of 2 mm. As a dose-volume histogram (DVH) parameters, we defined DV for absorbed dose in fraction *V*% (or *V* cm^3^) of the volume in the organ. In dose constraints for organ at risks (OARs), we determined *D*2% and mean absorbed dose (*D*mean) of the optic nerve, chiasm, and retina as upper limits of biologically equivalent dose at 2 Gy per fraction (EQD2) of 60 Gy and 45 Gy, respectively. In this case, we defined GTV of 0.35 cm^3^ using CT and MRI. Concerning a DVH parameter, *D*98% was 50.5 Gy for the GTV, *D*95% was 46.1 Gy for the PTV, and *D*2% and *D*mean were 2.0/0.7 Gy, 53.9/28.2 Gy and 4.9/1.2 Gy for the retina, optic nerve and chiasm, respectively.

**Fig. 3:**
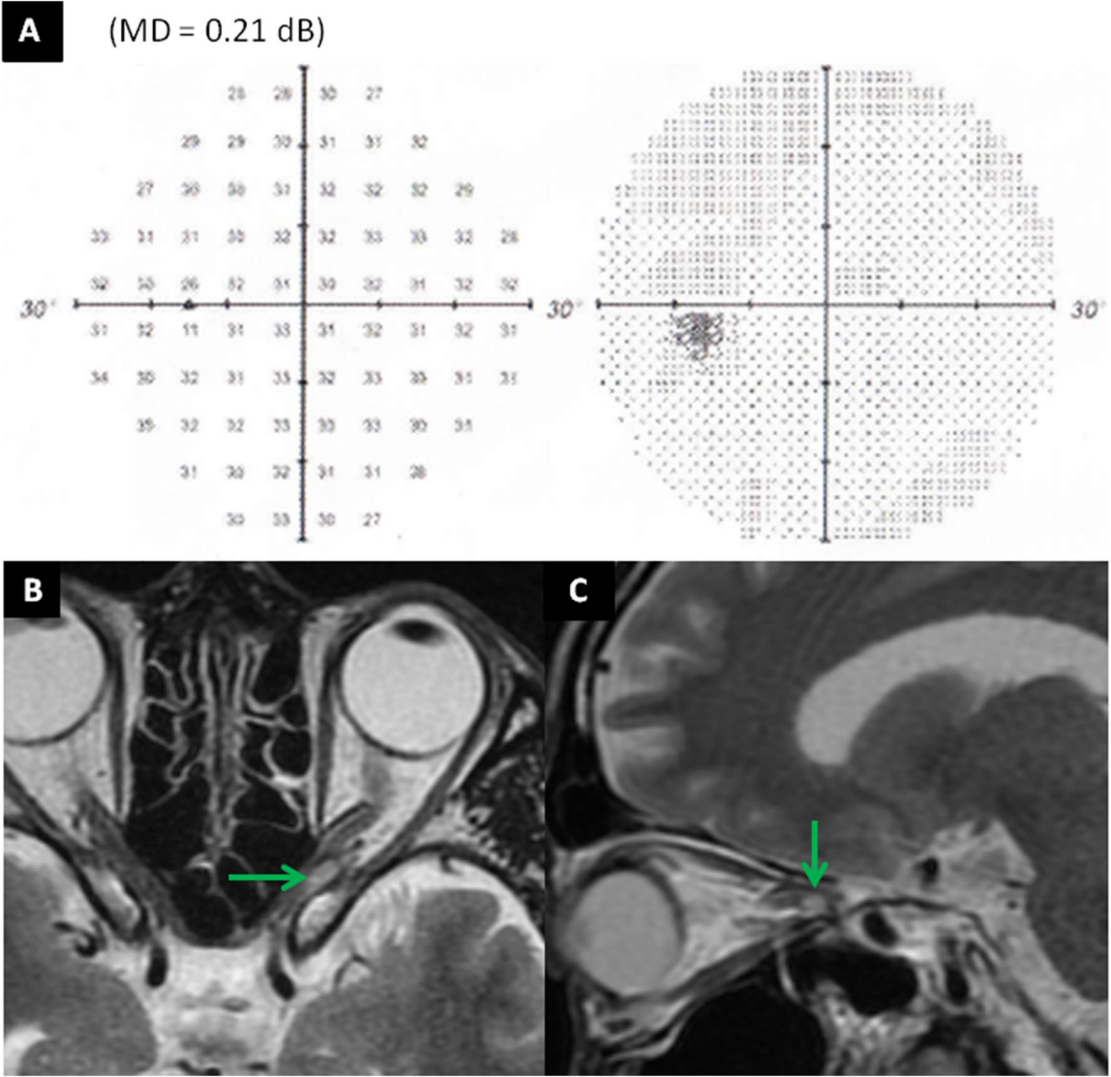
Humphrey automated perimetry showed complete disappearance of the visual field defect (**a**). Magnetic resonance imaging showed partial regression of the tumor (green arrow), with post-treatment dimensions of 6.5 × 3.5 × 5 mm (volume 0.1 cm^3^) 2.5 months after intensity-modulated radiotherapy completion (**b, c**)

We prospectively aimed to detect any early changes in VF occurring during the IMRT course using weekly HAP. An early change in VF was observed during the IMRT. The patient had a low-grade inferior altitudinal defect that has been detected by HAP before treatment. Repeated weekly HAP showed significant stepwise improvements in VF with a change in the mean deviation (MD) of sensitivity depression. At a dose of 46 Gy, HAP showed near-complete disappearance of the VF deficit with an MD of − 1.37 dB (*p* < 0.1: Fig. 1). Despite this marked improvement in VF, MRI showed little tumor reduction at a dose of 16 Gy (Fig. 2). Although HAP showed complete disappearance of the VF defect with an MD of 0.21 dB, MRI showed continued widening and winding of the ON, as well as only partial regression of the tumor with post-treatment dimensions of 6.5 × 3.5 × 5 mm (volume 0.1 cm^3^) 2.5 months after IMRT was completed (Fig. 3).

## Discussion

We observed an early improvement in visual function during a precision radiotherapy course in patients with ONSM [1]. Previously, Werner-Wasik described the interesting case of a patient who had shown early improvement in VA during a fractionated stereotactic radiotherapy (FSRT) course, as well as within 3 months of FSRT completion in a chapter under her charge [2]. The same author reported that such early effects were not rare, but that they were difficult to explain biologically. Jeremić supposed that the main reason for this occurrence was a combination of a decrease in radiation-induced edema and decompression of the functional nerve structures [2].

To this end, we prospectively tested a single patient’s VF using HAP at weekly intervals. HAP has been a longstanding standardized device for perimetry over 35 years or more [3], and HAP is a device to examine the VF automatically and can analyze the results statistically as well as quantitatively. Automated perimetry, i.e., HAP, offers obvious advantages to manual perimetry, i.e., Goldmann perimetry, in that stimulus presentation as well as the recording of patient responses can be standardized, leading to more reproducible result. MD is derived from the “total deviation” and represents the overall mean departure from the age-corrected norm [4]. A negative value indicates field loss, while a positive value indicates that the field is above average. A *p* value is provided if the global indices are abnormal. It provides a statistical representation of the population. For example, *p* < 2% means that less than 2% of the population have vision loss worse than measured.

No reports have yet described early improvement in VF, even though repeated weekly HAP tests have been carried out during precision radiotherapy courses. To our knowledge, this is the first report to do so. However, it is difficult to explain thoroughly why this stepwise improvement only occurs due to decompression in the ON caused in turn by macroscopic change in the tumor environment that occurs during early treatment.

In any case, it was generally agreed in the present study that functional improvements were not often followed by a significant decrease in tumor size upon imaging at various time points during the follow-up course. Moreover, any improvement in visual function could occur during the treatment course, not just later in the follow-up period [1].

Feasible mechanisms of compressive optic neuropathy (CON) can be attributed to the mass effect of direct or indirect compressive impacts with/without ischemia. In turn, these effects are caused by the compromised microcirculation of the optic nerve. For example, a pituitary tumor typically causes direct CON at the optic chiasm [5]; in contrast, the swollen extraocular muscles in Graves’ orbitopathy result in indirect CON at the orbit [6]. The latter responds successfully to corticosteroid pulse therapy, resulting in size reduction of the extraocular muscles [7]. Although optic nerve dysfunction in ONSM could be considered a direct CON, MRI showed no significant tumor reduction on treatment day 12 in the present case. Poor microcirculation perfusion might underlie this mechanism [8].

Harada et al. demonstrated that brain-derived neurotrophic factor signaling in the glia plays an important role in neural protection and regeneration, particularly in the conversion of Müller glia to photoreceptors [9]. We assume that the nodes of Ranvier, which are located every 0.05–1 mm along the ON sheath without myelin gaps [10], play an important role to respond VF defect in the precision radiotherapy. Micro-changes in the ON sheaths through the nodes of Ranvier have resulted in the random development, as well as the disappearance, of the dark points seen on HAP, which can manifest as a weave pattern in this case presentation. Even though MRI volumetry did not lead to significant tumor reduction, we observed rapid improvement in VF during the IMRT course.

We aimed to investigate the rapid improvement in visual function. To this end, we prospectively tested a single patient’s VF using HAP weekly intervals. The patient exhibited significant stepwise improvement in VF during an IMRT course.
